# Theoretical Bases for the Role of Red Blood Cell Shape in the Regulation of Its Volume

**DOI:** 10.3389/fphys.2020.00544

**Published:** 2020-06-09

**Authors:** Saša Svetina

**Affiliations:** ^1^Institute of Biophysics, Faculty of Medicine, University of Ljubljana, Ljubljana, Slovenia; ^2^Jožef Stefan Institute, Ljubljana, Slovenia

**Keywords:** Piezo1, Gárdos channel, mechanosensitivity, spectrin skeleton, curvature dependent protein–membrane interaction, cell to cell variability, osmotic fragility, negative feedback loop

## Abstract

The red blood cell (RBC) membrane contains a mechanosensitive cation channel Piezo1 that is involved in RBC volume homeostasis. In a recent model of the mechanism of its action it was proposed that Piezo1 cation permeability responds to changes of the RBC shape. The aim here is to review in a descriptive manner different previous studies of RBC behavior that formed the basis for this proposal. These studies include the interpretation of RBC and vesicle shapes based on the minimization of membrane bending energy, the analyses of various consequences of compositional and structural features of RBC membrane, in particular of its membrane skeleton and its integral membrane proteins, and the modeling of the establishment of RBC volume. The proposed model of Piezo1 action is critically evaluated, and a perspective presented for solving some remaining experimental and theoretical problems. Part of the discussion is devoted to the usefulness of theoretical modeling in studies of the behavior of cell systems in general.

## Introduction

The red blood cell (RBC) shape is, basically, assumed to depend on the cohesion and mechanical stability of its membrane (Mohandas and Chasis, 1993) and its volume to depend on the harmonized action of several different membrane pumps and channels that define the content of cytoplasm cations (Hoffmann et al., 2009). It is therefore considered that RBC shape and volume attain their physiological states independently of each other. The discovery that the RBC membrane includes a mechanosensitive channel, Piezo1, that has an effect on RBC dehydration (Murthy et al., 2017), indicated that RBC volume may also depend on membrane mechanics. Piezo1 acts through the activation of Gárdos channels by Ca^++^ ions that enter the cell when it is open (Cahalan et al., 2015). Recently we proposed a theoretical model in which it was postulated that Piezo1 cation permeability depends on an RBC discoid shape (Svetina et al., 2019). The model revealed the existence of a negative feedback loop that interrelates this shape with the RBC content of potassium ions and, thus, also with its volume. At the Monte Verita RBC meeting I reported about how predictions of the model were verified by utilizing the concepts developed in studies on RBC cell to cell variability (Svetina, 1982, 2017; Svetina et al., 2003). However, the model is a combination of these and several other concepts, together with views expressed previously in different theoretical studies on RBC shape and volume behavior. The present review will include the topics of these studies. This review is also motivated by the fact that the organizers of the Monte Verita meeting asked some senior participants to disseminate to newcomers to the field their research experiences. In this sense it will be rather subjective and thus largely concentrated on the work of our research group. The model discussed here is an example of the research approach by which, on the basis of theoretical analyses and exploitation of existing experimental data, it is possible to make predictions about the behavior of a treated system, thus providing new ideas as to how to advance the corresponding inquiries (Goldstein, 2018). We shall therefore discuss also some general capabilities of theoretical approaches in studies of cell processes.

To understand a given cell process it is necessary to identify the structural elements responsible and to provide a description of the mode of their operation. The corresponding theoretical studies are aimed at obtaining their structure–function relationship in a quantitative manner. This task is, in general, difficult, since cells are complex. The only way to make progress is frequently by analyses of mathematical models. In modeling it is usually necessary first to identify the structural level that is proper for the description of different aspects and for the function of a treated physiological process, and then to reveal its essential features on the basis of the simplest possible system. Models are, as a rule, built on the basis of a set of assumptions that can then be tested experimentally. When these assumptions are found to be correct, and it is thus possible to obtain model predictions by exact either analytical or numerical calculations, a model becomes a theory. The modeling approach should be distinguished from the use of mathematics in the analysis of experimental results and from simulations where, on the basis of the already established theory, the system’s behavior can be described mathematically in an exact manner. When modeling the behavior of whole cells it is advantageous to study those that are simple. RBCs, although composed of several thousand different molecules and ions, are, in some aspects, extremely simple. Basically, they are constituted by a concentrated hemoglobin solution enclosed by an essentially smooth membrane. Moreover, they also have a well-defined main function of carrying respiratory gasses. Therefore, and because of its availability, the RBC served, and still serves, as an ideal system for developing the principles of modeling structure–function relationships in cell systems in general (Lux, 2016).

This review will be focused on the bases on which we recently developed a model of the role of Piezo1 in the regulation of the RBC volume (Svetina et al., 2019). The aim is to help build a more thorough critical view on this model. The model was formed on the basis of several RBC and other research directions. It illustrates a circuitous nature of modeling approaches: the past theoretical studies on RBC shape have opened up some other research topics which have turned out to be relevant to studies of the regulation of RBC volume after the identification of the mechanosensitive protein Piezo1 (Coste et al., 2010) and the elucidation of its role in hereditary xerocytosis (Zarychanski et al., 2012). Briefly, some years ago we examined the possible osmotic states of RBC in dependence on the permeability state of its membrane (Brumen et al., 1979, 1981). In another study we presented (Svetina et al., 1982) a theoretical counterpart of the earlier proposed bilayer couple hypothesis of RBC shape transformation (Sheetz and Singer, 1974). This led us to formulate a general theory of shapes of vesicular objects with flexible membranes (Svetina and Žekš, 1989). This theory predicted that, among the possible stable shapes, some exhibit polar symmetry. We proposed that such shapes could serve as a mechanical origin of cell polarity, and also speculated that this could have been realized through curvature dependent interaction between membrane inclusions such as channels and pumps and the surrounding membrane (Svetina and Žekš, 1990; Svetina et al., 1990). We later derived a general phenomenological interaction term for the curvature dependent inclusion–lipid matrix interaction (Kralj-Iglič et al., 1999), and formulated the procedure for treating the mutual effects of the shape of a vesicular object and the lateral distribution of membrane inclusions on each other (Kralj-Iglič et al., 1996; Božič et al., 2006). These results gained significance because, in the meantime, several membrane proteins had been disclosed that were characterized by their membrane sensing and curvature forming capabilities (McMahon and Gallop, 2005; Zimmerberg and Kozlov, 2006). The curved Piezo1 structure (Ge et al., 2015; Guo and MacKinnon, 2017; Saotome et al., 2018; Zhao et al., 2018) indicates that it affects the shape of the surrounding membrane. A possible role of curvature dependent protein–membrane interaction in the process of mechanosensitivity has also been indicated (Svetina, 2015). The described broad modeling background thus seemed to be well suited also for analyzing different possible modes of Piezo1 operation in the regulation of RBC volume.

The review is organized as follows. The treated model (Svetina et al., 2019) will be described and commented in the last section (see section “Model of the Effect of RBC Discocyte Shape on RBC Volume and its Outlook”). The two intermediate sections will describe the model background. Section “The Mechanical and Thermodynamic Bases of RBC Shape and Deformability” deals with the RBC shape and deformability. In its first subsection it will be described how the initial theoretical studies of RBC shapes in which it was assumed that its membrane is laterally homogeneous led to a general theory of vesicular objects with flexible membranes. In the second subsection it will be shown how the difference between the predictions of this theory and the behavior of RBCs helps to understand the role of RBC membrane skeleton. Section “RBC Volume and Related Aspects of the Variability of RBC Population” will deal with the models of the regulation of RBC volume. Special attention will be devoted to the aspects of RBC population variability. It will then be shown how the results described in two previous sections can be combined in the model of the effect of Piezo1 on RBC volume. A critical review of this model will be given and some suggestions presented for the necessary future work. Throughout the review, the emphasis will be on the development of concepts, therefore it will be mostly presented in a descriptive manner. The corresponding equations and their derivation can be found in the cited literature.

## The Mechanical and Thermodynamic Bases of RBC Shape and Deformability

The function of RBC as the carrier of respiratory gasses led to its adoption throughout evolution of numerous specific mechanical and thermodynamic properties. In the absence of external forces, the normal RBCs of most vertebrates assume the shape of a disk that involves, at its poles, the presence of symmetrically indented dimples. RBCs are deformable, e.g., under microcirculatory flow conditions, at sufficiently high shear stress, deform into rolling stomatocytes and, finally, adopt polylobed shapes (Lanotte et al., 2016). An important factor that allows for these shape transformations is that RBC occupies only about 60% of the volume that a cell could at a given area of its membrane. This property of RBCs is conveniently quantified in terms of the reduced volume (*v*) defined as the ratio between the RBC volume (*V*) and the volume of the sphere with the same membrane area (*A*):

(1) $$v=3V/4\pi R_{\text{s}}{}^{3}$$

where *R*_s_ = (*A*/4π)^1/2^. The mechanism for the establishment of RBC volume will be dealt with in section “RBC Volume and Related Aspects of the Variability of RBC Population.” Here our focus is on how, at a given value of *v*, RBC shape and its deformation depend on the mechanical properties of its membrane. RBC membrane is composed of a lipid bilayer occupied densely by membrane integral proteins, and the underlying membrane skeleton, a two-dimensional pseudo-hexagonal network with actin based protein complexes as nodes and spectrin tetramers as bonds. The bilayer and the skeleton are linked by chemical bonds between spectrin and integral membrane proteins band3 and glycophorin C, via ankirin and actin complexes, respectively (Mohandas and Gallagher, 2008; Lux, 2016). The RBC membrane differs from those of most other eukaryotic cells in that it has no cytoplasm reservoirs and has therefore a smooth appearance. Because of a relatively large value of the compressibility modulus of the bilayer it is, laterally, practically incompressible. RBC mechanical behavior depends crucially on the characteristic of its membrane that its three layers, the two leaflets of the bilayer and the underlying skeleton, can slide, one over the other. The bilayer resists bending of the membrane and the skeleton to exhibit shear deformation. The first reasonable models of RBC shape behavior were based on the assumption that their shapes correspond to the minimum of membrane bending energy. In the subsequent subsection it will be revealed how, out of these models, a theory of shapes of simple vesicular objects such as phospholipid vesicles developed, and how the stability of shapes depends on the elastic properties of multilayered membranes. In the second subsection it will be shown how the comparison between the predictions of this theory and the behavior of RBC helps the different roles of its membrane skeleton to be understood.

### Interpretation of RBC and Vesicle Shapes on the Basis of Membrane Bending

Red blood cell shape has been treated by assuming its membrane to be a single, thin, laterally homogeneous mechanical entity. RBC membrane can, at its reduced volume *v* of about 0.6, take up an infinite number of shapes, exhibiting different values of the total membrane bending energy (*W*_b_) that can be for symmetrical bilayer obtained by the integral of the square of the mean membrane curvature (*H* = (*C*_1_ + *C*_2_)/2 where *C*_1_ and *C*_2_ are the principal membrane curvatures) over the whole membrane area expressed as

(2) $$W_{\text{b}}=2\,k_{c}\,\int H^{2}\,dA$$

with *k*_c_ membrane bending constant. In general membrane bending energy involves also a contribution due to Gaussian curvature (*K* = *C*_1_*C*_2_) (Helfrich, 1973). However, because the integral of *K* over the membrane area is for a given membrane topology constant this term will be in further discussions here ignored. Canham (1970) looked for the minimum of *W*_b_ (Eq. 2) and found that, at *v* = 0.6, the shape is a discoid. He assumed that the membrane has zero energy when it is flat and took into consideration that the membrane has no lateral shear, i.e., that it behaves laterally as a two-dimensional liquid. Helfrich (1973) generalized the expression for membrane bending energy by assuming that the membrane may have, due to transmembrane asymmetry, zero energy when it is bent to its spontaneous curvature (*C*_0_). Deuling and Helfrich (1976), by applying their “spontaneous curvature model,” obtained by minimizing the Helfrich’s (1973) expression of membrane bending energy at given reduced volumes and reduced values of the spontaneous curvature (*R*_s_*C*_0_) beside the discocyte also several other shapes including cup shaped stomatocytes. At about the same time Sheetz and Singer (1974) introduced the “bilayer couple hypothesis” based on the evidence that RBCs change shape under conditions of asymmetric changes of the areas of the outer and inner leaflets of the membrane bilayer. They showed that, by adding a drug (chlorpromazine) that intercalates into the inner monolayer of the RBC bilayer, the discocyte transforms into a cup shape (stomatocyte) whereas drugs intercalating into the outer layer cause shape transformation into a spiculated echinocyte. In a theoretical treatment of the bilayer couple hypothesis, RBC shapes (which were defined in terms of the finite number of geometrical parameters) were obtained by minimization of the membrane bending energy at a fixed difference between the areas of the outer and inner leaflets of the bilayer (Svetina et al., 1982). It was implied that this area difference (Δ*A*) constitutes a convenient single parameter whose continuous decrease causes the shape to be transformed from discocyte to stomatocyte in a continuous manner. This result was confirmed and, also, further explored by an exact variational procedure for minimizing membrane bending energy under the constraints of constant membrane area, cell volume, and area difference (Svetina and Žekš, 1989). While bilayer couple hypothesis represents for some aspects of RBC shape transformations a useful workable model, it also turned out to be a strict theory for shapes of simple vesicular objects defined as a liquid interior enclosed by a flexible membrane. For students of RBC shape behavior and deformability it is useful to be familiar with the basic results of this theory because knowledge of its predictions may help to distinguish which aspects of RBC behavior depend on properties of its bilayer and which on its other structural features.

**FIGURE 1:**
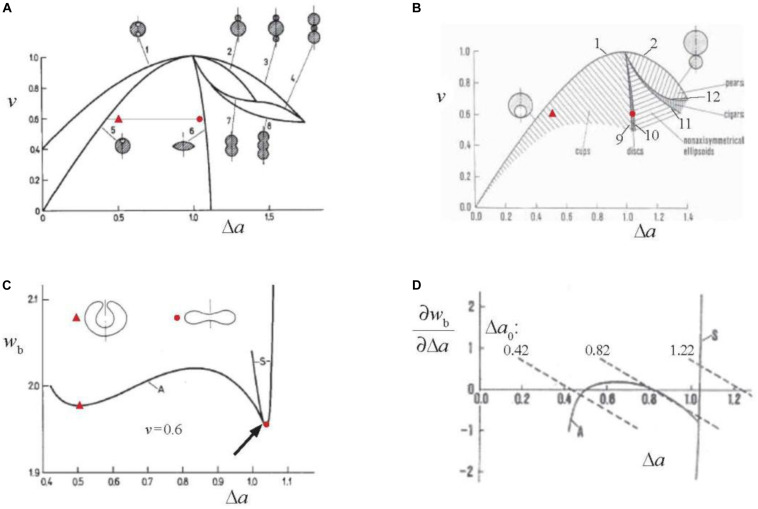
Demonstration of basic features of the bilayer couple theories of vesicle shapes [**(A–C)** of “strict” and **(D)** of “generalized”]. **(A)** *v*(Δ*a*) dependences of limiting shapes which correspond to the extreme values of *v* at a given value of Δ*a* (lines 1 to 8) plotted in the *v* – Δ*a* shape phase diagram (Svetina and Žekš, 1989). Examples of corresponding shape cross-section are also shown. The dotted horizontal line at *v* = 0.6 indicates the range of Δ*a* values for which minimal membrane bending energy is presented in **(C)**. Red point indicates the phase diagram location of a discocyte and red triangle of a characteristic stomatocyte. **(B)** Stable vesicle shapes in the central part of the *v* – Δ*a* shape phase diagram. Lines 9–12 are symmetry breaking lines. Marked areas represent the parts of the shape phase diagram where there are no contacts between different regions of the membrane. The meaning of red points is the same as in **(A)**. **(C)** Membrane bending energy *w*_b_ (defined relative to the bending energy of the sphere which is 8π*k*_c_) calculated at *v* = 0.6 for the Δ*a* values indicated by the dotted line in **(A)**. Line S shows bending energy of disk shapes and line A of the cup shapes. The arrow indicates the point of the symmetry breaking of the disk shape as predicted by Svetina and Žekš (1989). Shown are also contours of cross-sections of a discocyte (red point) and a stomatocyte (red triangle) and it is indicated which are their Δ*a* values. **(D)** The dependence of the partial derivatives of the bending energies S and A presented in **(C)** by the area difference Δ*a*. The dotted lines present the right hand side of Eq. 4 for the indicated values of Δ*a*_0_ and *k*_r_/*k*_c_ = 3 (reprinted with permission from Svetina, 1998).

The bilayer couple theory (Svetina and Žekš, 1989) predicts that vesicle shapes depend on only two geometrical parameters, the reduced volume *v* and the reduced area difference Δ*a* (defined as the ratio between Δ*A* and its value for the sphere which is 8π*hR*_s_ with *h* the distance between the neutral surfaces of the bilayer leaflets and *R*_s_, as already defined, the radius of the sphere). The geometrical meaning of Δ*a* is that it is also equal to the integral of the reduced mean membrane curvature (*R*_s_*H*) over the membrane surface. Vesicle shapes can be grouped into classes that occupy different parts of the *v* – Δ*a* (or Δ*a* – *v* as used in Seifert et al., 1991) shape phase diagram. The shapes belong to a given class if they have the same symmetry and if, by continuously changing Δ*a* and/or *v*, they are changing continuously. The sense of such shape classification is illustrated in Figures 1A–C. There are two types of shape class boundaries. One type comprises shapes obtained by variational search of the extreme values of the reduced volume *v* at a fixed value of the reduced area difference Δ*a* (Figure 1A). They are composed of spheres or spherical parts with only two possible values of their radius (Svetina and Žekš, 1983, 1989). For example, lines 1 and 6 are boundaries of shape class to which belongs the discocyte (located in the minimum of the bending energy curve “S” in Figure 1C). All these shapes are axisymmetric and involve equatorial mirror symmetry. The second type of shape class boundaries are symmetry breaking lines (lines 9 to 12 in Figure 1B). For example, the class of cup (stomatocyte) shapes is, on one side, bounded by the limiting shape (line 5 in Figure 1A) and, on the other side, by the symmetry breaking line (line 9 in Figure 1B) that connects the points at which the equatorial mirror symmetry of disk shapes breaks down (shown by an arrow in Figure 1C) at all reduced volumes. Notably, the class of non-axisymmetric (ellipsoidal) shapes is bounded by symmetry breaking lines on both of its sides (lines 10 and 11 in Figure 1B) at which disk and cigar shapes, respectively, break down their axial symmetry (Heinrich et al., 1993). As demonstrated by curves A and S in Figure 1C, classes overlap. Only the shape with the lowest bending energy is stable. Figure 1B shows which shapes are stable within the presented central part of the *v* – Δ*a* shape phase diagram. Red point and triangle in Figures 1A–C indicate where in the *v* – Δ*a* shape phase diagram are located the discocyte and typical stomatocyte, respectively. The significance of the bilayer couple theory is that it predicts all possible shapes of vesicular objects with laterally homogeneous membranes. If a vesicle shape differs from any of these shapes it means that there are external forces acting on it (Svetina and Žekš, 1996) or that its membrane is laterally inhomogeneous (Božič et al., 2006).

The described predictions of the bilayer couple model are strictly only valid if the two equally composed leaflets of a bilayer are incompressible. In reality they are compressible and therefore it has to be taken into account that, in general, in a given shape, they might be deformed differently, for example in that the area of one is extended and of the other compressed. In such cases the reduced area difference (Δ*a*) differs from analogously defined equilibrium (preferred) area difference (Δ*a*_0_) which corresponds to the situation where leaflets are neither extended nor compressed. The bilayer thus exhibits, in addition to the already defined bending energy (Eq. 2), also the non-local bending energy (*W*_k_) (Evans, 1980), termed also as area difference elastic term (Miao et al., 1994), expressed in its reduced form (*w*_k_ = *W*_k_/8π*k*_c_) as

(3) w=kk(Δa-Δa)0r/22

where *k*_r_ is the non-local bending constant. The derivation and consequences of non-local bending energy were comprehensively reviewed in Svetina and Žekš (2014). Briefly, in the generalized bilayer couple model, vesicle shapes correspond to the minimum of the sum of the bending (Helfrich, 1973) and non-local bending energies. The shape equation to be solved is the same as in the limit of the strict bilayer couple model so the shapes obtained are the same. However, not all of them are necessarily stable. Why it is so is demonstrated by Figure 1D. The minimization of the sum of the two bending energies with respect to Δ*a* gives rise to the requirement

(4) ∂w/b∂Δa=-(k/rk)c(Δa-Δa)0

The solution of Eq. 4 for its unknown Δ*a* can, for a given value of Δ*a*_0_, be obtained graphically as a point on the graph of Figure 1D where a dashed curve (right hand side of Eq. 4) crosses one (either S or A) of the ∂*w*_b_/∂Δ*a* curves (left hand side of Eq. 4). The number of solutions of Eq. 4 at given Δ*a*_0_ depends on the slope of dashed curves that is proportional to the ratio *k*_r_/*k*_c_. There is only one solution if this slope is steeper than that of the largest derivative by Δ*a* of the function ∂*w*_b_/∂Δ*a* of asymmetrical shapes (A) which is at the symmetry breaking point (Figure 1C). A vesicle can thus attain all possible shapes predicted by the strict bilayer couple model. At values of *k*_r_/*k*_c_ that are smaller than above defined critical value of ∂*w*_b_/∂Δ*a* there are, for some values of Δ*a*_0_ (e.g., 0.82 in Figure 1D), three solutions of Eq. 4. The shape at the middle value of Δ*a* is not stable. Consequently there is, e.g., at continuously decreasing value of Δ*a*_0_, a discontinuous shape transformation from the Δ*a* at a cross-section of a dotted line with the curve S to the smaller Δ*a* at which this line crosses the curve A. The shapes of the strict bilayer couple model that correspond to the intermediate Δ*a* values are not stable. Possible stable shapes of the generalized bilayer couple model are thus defined by the 3-dimensional *v* – Δ*a* – *k*_r_/*k*_c_ shape phase diagram. The example of the cross-section of this diagram is for *v* = 0.85 shown in Figure 12 of Svetina and Žekš (1996). The slope of the ∂*w*_b_/∂Δ*a* curve at the symmetry breaking point is at *v* = 0.6 close to – 3. The estimated ratio *k*_r_/*k*_c_ for RBC membrane is about 2 (Hwang and Waugh, 1997) which indicates that the discocyte–stomatocyte transition is discontinuous. The described reasoning can be generalized straightforwardly to cover also the effects of transmembrane asymmetry characterized by membrane spontaneous curvature *C*_0_. *C*_0_ and Δ*A*_0_, in spite of having different physical background affect the shapes of vesicular objects with bilayer membranes in a similar manner. The stationary shapes obtained by solving the shape equation are the same as in the strict bilayer couple model if for the reduced equilibrium area difference is taken an effective one defined as

(5) Δa=0,e⁢f⁢fΔa+0ck0/c2kr

It has to be noted that in this case the region of stable shapes in the generalized shape phase diagram *v* – Δ*a*_0,eff_ – *k*_r_/*k*_c_ depends on the relative contribution to Δ*a*_0,eff_ of Δ*a*_0_ and *c*_0_. It is because the energy term due to Δ*a*_0_ (Eq. 3) involves Δ*a*^2^ whereas the energy term due to *c*_0_ is a linear function of Δ*a*. Therefore the discontinuous transition indicated in Figure 1D occurs at shifted Δ*a* values, such that at increasing the relative contribution of *c*_0_, the region of stable shapes is diminishing. The limit *k*_r_/*k*_c_ = 0 represents the spontaneous curvature model of Deuling and Helfrich (1976). A full description of which shapes are stable in this limit was presented by Seifert et al. (1991) (reviewed in Seifert, 1997). The spontaneous curvature model also applies in the case that due to transmembrane lipid transport the bilayer relaxes into the state with Δ*a*_0_ = Δ*a*. The relaxation time for this process was estimated to be 8 min (Raphael and Waugh, 1996) or even much less (Svetina et al., 1998).

### Effects of Compositional and Structural Features of the RBC Membrane

Red blood cell membrane is, compared to phospholipid membranes, complex. Its bilayer part is crowded with integral membrane proteins such as band3 that is involved in RBC’s function of carrying carbon dioxide, different pumps and channels that take care of the establishment of RBC volume, and many other proteins serving in its protection (Mohandas and Gallagher, 2008). As already noted, it contains, on its cytoplasmic side, a spectrin based membrane skeleton which is the main element that accounts for how RBC shape behavior differs from that of simple phospholipid vesicles. In this respect we here discuss skeleton shear elasticity, its role in the formation of RBC shape, and its possible effects on the lateral distribution of integral membrane proteins and their lateral diffusion.

**FIGURE 2:**
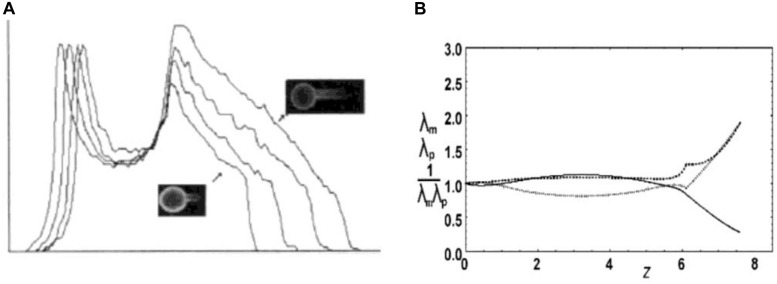
Deformation of RBC skeleton. **(A)** Experimental evidence for the deformation of the membrane skeleton when RBC ghost is aspirated into a medium size pipette (*R*_p_ ≈ 2 μm) (from Discher et al., 1994; reprinted with permission from AAAS). The skeleton density profiles along the projection of four aspirated RBC ghosts are shown, obtained by measuring fluorescein-phalloidin-labeled actin. Relevant for the present discussion is the decrease of the intensity along the aspirated part of the ghosts (arrows). **(B)** Skeleton extension ratios along parallels *λ*_p_ (dashed) and along meridians *λ*_m_ (dots) and the density 1/*λ*_p_*λ*_m_ (relative to its mean value; full line) calculated according to the described model (Svetina et al., 2016) for the RBC aspirated into medium size pipette **(A)** (reprinted with permission from Švelc and Svetina, 2012). The pipette is on the right. The initial shape with, presumably, homogeneous skeleton distribution is a discocyte.

Red blood cell membrane exhibits shear elasticity. Because its bilayer part can be considered as two-dimensional liquid, the shear elasticity can be ascribed solely to its membrane skeleton which is a two-dimensional pseudo-hexagonal network of spectrin tetramers as bonds and acting filaments as nodes. To understand the skeleton behavior it is crucial to realize that RBC membrane deformation may cause an alteration of local skeleton densities while the density of the lipids remains the same, as was observed by measuring skeleton lateral distribution in RBC partially aspirated into the micropipette (Discher et al., 1994; Discher and Mohandas, 1996; Figure 2A). These results imply that skeleton nodes shift their position relative to the bilayer and that the bonds deform elastically. This is possible because the bilayer integral proteins to which the skeleton is anchored can move laterally in the plane of the bilayer. The observed changes of skeleton density indicate that the extension ratios (*λ*_i_, the ratio between the final length and the initial length of the deformed skeleton material in the i-th direction) may reach a value of about 3, which corresponds to a fully extended spectrin tetramer of length ∼ 200 nm. Skeleton deformation may be described by shear and area compressibility deformational modes (Mohandas and Evans, 1994). The area compressibility elastic modulus of the RBC skeleton is estimated at 26 μN/m (Svetina et al., 2016), which is about four orders of magnitude less than that of the whole RBC membrane determined to be 0.29 N/m (Evans et al., 1976). Consequently, the deformed skeleton redistributes over the RBC membrane at its practically constant total area *A*. Different parts of RBC skeleton are kept together by non-covalent bonds which can break and reform, indicating the possibility that it is plastic. The undeformed state of the skeleton may thus depend on the RBC’s history and is in general not well defined.

Theoretical modeling of the RBC skeleton is developing in several different directions (e.g., Discher et al., 1998; Fedosov et al., 2010; Peng et al., 2010; Svetina et al., 2016). The present discussion will focus on the type of models aimed at making a distinction between skeleton deformation due to the change of the shape of the cell membrane and that due to its mechanical properties. In this respect Mukhopadhyay et al. (2002) applied in their model of the RBC membrane the concept of a mapping function defined in terms of the dependence of the original position of a skeleton element on its position in the deformed state. They used this concept in their studies of the effect of the skeleton on RBC shapes (see below). For axisymmetric shapes it is possible to express the mapping function as *s*_0_(*s*) where *s*_0_ is the arc-length distance from the cell pole to a given contour point of the original skeleton state and *s* the corresponding distance of the deformed state. Mathematically, the dependence *s*_0_(*s*) is sought at which the sum of the skeleton energy and the membrane bending energy is minimal. We treated a simplified version of this problem by studying cases in which the deformed shape is defined by rigid walls and therefore there is no need to consider the bending energy (Svetina et al., 2016). Examples of such deformations are RBC aspiration into medium sized (with respect to the RBC size) (Figure 2A) and narrow (∼ 1 μm) pipettes. Following Discher et al. (1998) we also assumed that the main contribution to the skeleton energy is the energy of spectrin bonds. Instead of using for bond energy a more realistic flexible chain model (Discher et al., 1998), we described this energy by a harmonic potential which is a good approximation at sufficiently small deformations (Figure 8 in Svetina et al., 2016). The energy of the deformed skeleton was determined in the mean field approximation. In this simple model the skeleton deformation is the same for any value of the bond strength. This means that the most important factor for the observed change in skeleton lateral distribution is the changed cell geometry. For axisymmetric shapes it is, to some extent, possible to reason about the effect of changed geometry in qualitative terms. When a patch of the skeleton that is at a distance *r*_0_ from the axis moves in the deformed state to the distance *r* from the axis, its extension along the parallels is *λ*_p_ = *r*/*r*_0_. The corresponding compression (in case that *r* < *r*_0_) exerts a tendency to make *λ*_m_ larger than 1. The local magnitudes of the extensions along meridians are restricted by the requirement that the total skeleton area is constant. The effect of this requirement cannot be visualized so clearly; however, it can still be concluded that the skeleton prevents shape changes with significant changes of the distances of the membrane from the axis. In Figure 2B, as an example, is shown by this model predicted deformation of RBC discocyte aspirated into a medium sized pipette.

Red blood cell shape behavior differs qualitatively from that of phospholipid vesicles in the region of the *v* – Δ*a* shape phase diagram, where prolates are the typical equilibrium shapes of vesicles with simple membranes (e.g., dumb-bells and pears) with their limiting shapes involving external buds (Figures 1A,B). In contrast, RBC shapes are, in the respective Δ*a* region, echinocytic (reviewed in Svetina et al., 2004). Mukhopadhyay et al. (2002) studied the formation of echinocytes on the basis of continuum mechanics by searching for the minimum of the sum of the local and non-local bending energies of the bilayer, and the stretching and shear elastic energies of the membrane skeleton. The bending energy of the spiculated membrane is larger than that of the dumb-bell shape. However, the echinocyte can be understood to be more stable since the deformation of the skeleton into the geometry of a dumb-bell would, because of large changes of the distances from the axis, require a much larger increase of the skeleton energy than that for its transformation into a quasi-spherical echinocyte (neglecting that some skeleton albeit with decreased density is also present in its spicules). The formation of echinocytes has a physiological advantage by preventing the occurrence of shapes with large external buds that form in simple vesicles at increased values of Δ*a* (c.f. line 3 in Figure 1A). The probable pinching off of such buds in the turbulent blood flow would increase RBC reduced volume considerably, thus diminishing its deformability. It should be noted that when RBC changes its shape from discocyte to stomatocyte, e.g., due to possible decrease of membrane Δ*a*_0_, the skeleton is not deformed so much because these two shapes are both oblate and the distances of skeleton elements from the axis in them do not differ appreciably. Therefore, the behavior of RBC shape in the “oblate” region of the *v* – Δ*a* shape phase diagram does not differ essentially from the behavior of simple phospholipid vesicles. In cases where the buds are internal (c.f. lines 1 and 5 in Figure 1A) there is also no danger that they would be pinched off.

**FIGURE 3:**
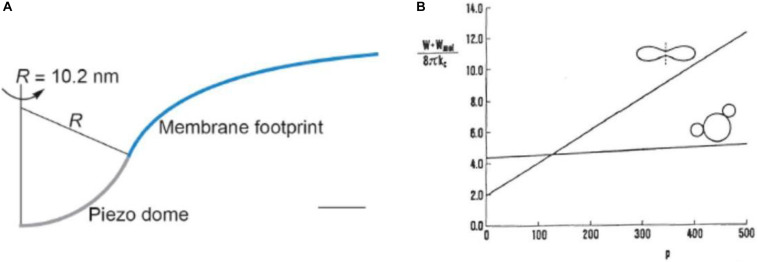
Illustrations of effects of protein–membrane interaction. **(A)** The shape of the Piezo1 membrane footprint shown as the cross-section of the mid-bilayer surface and its intersection with the Piezo1 dome; scale bar: 4 nm (reprinted from Haselwandter and MacKinnon, 2018, held under CC-BY 4.0 license). **(B)** The sum of the membrane bending energy and the distributional free energy of mobile membrane inclusions calculated for the disk shape and for the shape that involves two buds for a cell with the reduced volume 0.6 in the dependence of the number of inclusions *p* (in some reduced units) (reprinted with permission from Svetina et al., 1996).

Another physiological role of the RBC membrane skeleton is that of the prevention of formation of risky budded shapes due to lateral segregation of its integral proteins. Membrane embedded proteins interact with the surrounding membrane when their intrinsic principal curvatures differ from those of the membrane at their location. For example, when the drastically curved protein Piezo1 (Guo and MacKinnon, 2017) is embedded in a flat phospholipid membrane it is predicted that it will cause the membrane in its surroundings to form a kind of dome-shaped invagination (Figure 3A, from Haselwandter and MacKinnon, 2018). It is possible to treat such a protein–membrane interaction in terms of a phenomenological expression that takes into account the fact that there is a mismatch between the intrinsic principal curvatures of membrane inclusion (e.g., a protein) and those of the membrane. The general expression for the corresponding energy term, in the limit of a rigid inclusion surface, is conveniently written as (Kralj-Iglič et al., 1999)

$$W_{c\,u\,r\,v,j}=\frac{\kappa_{j}}{2}\,{\left(H-H_{P,j}\right)}^{2}$$

(6) $$+\frac{\kappa_{j}^{{}^{*}}}{2}\,\left[\Delta \,H^{2}-2\,\Delta \,H\,\Delta \,H_{P,j}\,\cos\left(2\,\omega_{j}\right)+\Delta \,H_{P,j}^{2}\right]$$

where *H*_P,j_ = (*C*_1,P,j_ + *C*_2,P,j_)/2 is the mean principal intrinsic curvature of the transmembrane part of the inclusion and Δ*H*_P,j_ = (*C*_1,P,j_ − *C*_2,P,j_)/2 is a measure of the difference between its two principal curvatures. *κ*_j_ and *κ*_j_^∗^ are independent interaction constants. The angle *ω*_j_ defines the mutual orientation of the coordinate systems of the intrinsic principal curvatures of the inclusion and the principal curvatures of the membrane. One consequence of such interaction term is curvature sensing, meaning that mobile membrane proteins, due to curvature dependent interaction energy term, accumulate in membrane regions where this mismatch is small and are depleted from regions where it is large. For example, it is reasonable to expect that it is more probable for the Piezo1, due to its curved structure, to reside in regions of RBC discocyte poles (dimples) than on its equator. The second possible consequence of the curvature dependent protein–membrane interaction is its effect on shape which, for a membrane with mobile proteins, corresponds to the minimum of the sum of their distributional free energy and the bending energy of the membrane (Božič et al., 2006). The effect of inclusions on RBC shape depends on their number. Using a model study (Svetina et al., 1996), it was shown that, for the discocyte, the minimum system’s free energy depends on the number of inclusions in a much stronger manner than in that for a budded shape (Figure 3B). If this number is sufficiently small the bending energy prevails and the stable shape remains to be the disk. However, above a certain critical value, the budded shape has a lower free energy. RBC membrane contains about 10^6^ band3 proteins which could constitute a potential danger for the stability of the RBC if most of them were not linked to the skeleton.

Red blood cell membrane proteins that are not linked to the skeleton can, upon the deformation, redistribute over the membrane with a time constant that depends on their diffusion coefficient. The latter can be smaller than in a vesicle because of the corralling effect of the spectrin skeleton (Tomishige et al., 1998). A drastic reduction of the diffusion constant can be expected for Piezo1 because the membrane indentation that it causes (Figure 3A). It just about fits into the triangle formed by the three spectrin tetramers, with each of them having in the RBC’s resting state the length of about 70 nm.

## RBC Volume and Related Aspects of the Variability of RBC Population

Red blood cell membrane is, as those of most mammalian cells, well permeable for water. Therefore the RBC’s water content, and thus also its volume, depend on its content of osmotically active substances and on the external tonicity (Hoffmann et al., 2009). Hemoglobin, the main protein constituent of the RBC cytoplasm, cannot cross its membrane, so therefore the RBC is under osmotic stress. In RBCs of many species, their reduced volume is, by virtue of the active pump-leak system for monovalent cations K^+^ and Na^+^, nevertheless kept at about 0.6. These cations are pumped by Na^+^/K^+^- ATPase which actively expels three sodium ions and takes in two potassium ions (Tosteson and Hoffman, 1960; Kay and Blaustein, 2019). The consequent higher cell concentration of K^+^ and lower concentration of Na^+^, both relative to their concentration in the environment, cause fluxes of these two cations in the direction of their concentration gradients. The pumping and leaking of K^+^ and Na^+^ eventually leads to the stationary volume level. There are several channels/transporters involved in the passive leakage of these two cations. The results of many studies of their action and of the data on cation pumping made it possible to formulate realistic mathematical modeling of RBC volume regulation (Lew and Bookchin, 1986; Armstrong, 2003; Ataullakhanov et al., 2009). K^+^ and Na^+^ attain their stationary value in a time scale which is several orders of magnitude larger than that of water and also of univalent anions Cl^–^ and HCO_3_^–^ which can thus be treated at correspondingly short time scales as being in quasi-equilibrium between the inside and outside solutions. Because hemoglobin is charged, this equilibrium can be described by models that involve a version of the Donnan equilibrium in which cations do not exchange (Brumen et al., 1979; Freedman and Hoffman, 1979).

**FIGURE 4:**
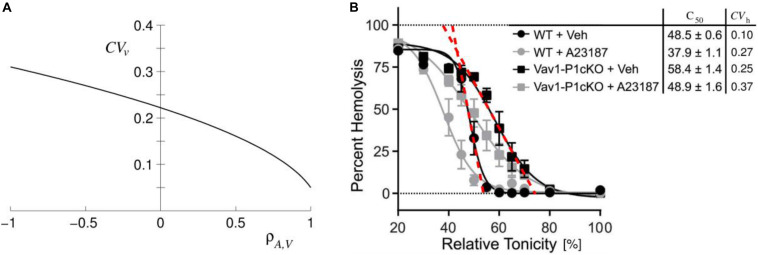
Illustrations of consequences of the correlation between RBC area (*A*) and volume (*V*). **(A)** Coefficient of variation of RBC reduced volume (*CV*_v_) in dependence on the correlation coefficient *ρ*_A,V_ obtained for the values of coefficients of variations of RBC volume and membrane area to be 0.12 and 0.13, respectively (reprinted with permission from Svetina et al., 2019). *CV*_v_ at *ρ*_A,V_ = 0.96 is about 0.06. **(B)** Evidence for the role of Piezo1 based regulation of RBC volume on the mouse RBC *V* – *A* correlation. Cahalan et al. (2015) measured osmotic fragility of normal (WT) and of Piezo1 knocked out (Vav1-PicKO) mouse RBC in the absence (+Veh) and presence (+A23187) of Ca^2+^ ionophore A23187 (reprinted with permission from Svetina et al., 2019). In Figure 5 of Svetina et al. (2019) we added to Figure 3D of Cahalan et al. (2015) the column of the corresponding coefficients of variation (*CV*_h_) obtained from steepness of osmotic fragility curves as indicated by red dashed lines.

The issue here is the extension of already established models of RBC volume regulation that take into consideration the role of Piezo1 and Gárdos channels (Svetina et al., 2019). The predictions of the proposed model were supported by studies on properties of RBC population cell to cell variability. RBCs, in otherwise homogeneous RBC population, are known to be variable with respect to many of their measurable parameters such as cell volume, membrane area, hemoglobin content, density, etc. RBCs vary because of the variable properties of their precursors and because, throughout their lifespan, they are releasing nanovesicles (Westerman and Porter, 2016). In general, cells of the same kind are presumably organized in an identical manner, meaning that their state is defined by the same physical-chemical processes. On the basis of this assumption it can be deduced that, if there are some strict algebraic relations between the parameters that define the state of a single cell, there are also relationships between the parameters that measure the variability of these parameters, e.g., coefficients of variation and correlation coefficients. This notion has been confirmed by analysis of relations between standard deviations and correlation coefficients extracted from different single cell measurements of RBC volume, membrane area, density, and hemoglobin and cation contents (Svetina, 1982). Moreover, this analysis also revealed the possibility that an RBC involves a strict relationship, of at that time unknown origin, between membrane area and hemoglobin and cation contents. The analogous conclusion was later, by a different approach, obtained by Lew et al. (1995). The basic source for such relation was realized to be the strong correlation between RBC volume and membrane area. Simultaneous single cell determinations of these two cell parameters yielded for the corresponding correlation coefficient a value of *ρ*_A,V_ ∼ 0.97 (Canham and Burton, 1968) and of ∼ 0.96 (Gifford et al., 2003). The strong correlation between *V* and *A* is reflected in the fact that the coefficient of variation of the reduced volume is only about one half of the coefficients of variation of *V* and *A* (Figure 4A). It also explains the narrowness of the coefficient of variation for the hemolytic osmotic pressure presented in Figure 2 of Lew et al. (1995). With respect to the modeling of the regulation of the RBC volume it can be concluded that one of the model parameters should be membrane area. Recent evidence indicates that the process responsible for *V* – *A* correlation involves Piezo1 (Cahalan et al., 2015). Namely, in Piezo1 knockout mice, and in the case where Piezo1 action of opening Gárdos channels was overruled by the Ca^2+^ ionophore A23187, this correlation was lost (Figure 4B).

## Model of the Effect of RBC Discocyte Shape on RBC Volume and Its Outlook

The fact that RBC dehydration in hereditary xerocytosis can be caused by malfunctioning of a mechanosensitive protein Piezo1 indicates that RBC volume may also depend on mechanical properties of RBC membrane. Piezo1 system appeared to represent a relatively independent module of the otherwise complex regulation of RBC volume, and could thus be considered as an ideal candidate for application of the modeling approach. In the model under consideration (Svetina et al., 2019) we chose, for its elements that are crucial for the action of Piezo1, the sensing of the membrane curvature through curvature dependent inclusion–membrane interaction (Svetina et al., 1990; Kralj-Iglič et al., 1996, 1999), and the assumption that the mechanical properties of an RBC membrane affect its volume through the dependence of Piezo1 Ca^++^ permeability on RBC discocyte shape. These ideas were supported by curved structure of the Piezo1 trimer, evidenced by its structural studies (Ge et al., 2015; Guo and MacKinnon, 2017; Saotome et al., 2018; Zhao et al., 2018) and, indirectly, also by the altered RBC membrane cation permeability when stressing the cells by a distorting device (Kuchel and Shishmarev, 2017) or by a flow through a capillary constriction (Cinar et al., 2015; Danielczok et al., 2017). Here we shall first outline which of the topics presented in the previous two sections formed the basis of this model. Then we shall evaluate its outcome, define its deficiencies and indicate some model implications to improve the understanding of RBC Piezo1 action by further experimental and theoretical studies.

**FIGURE 5:**
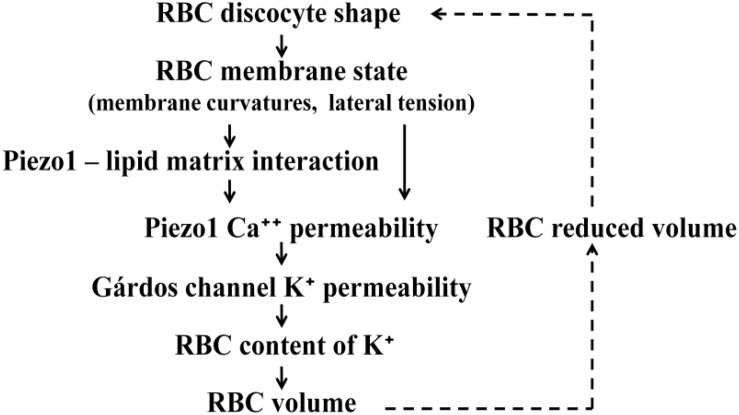
Schematic presentation of processes involved in the effect of RBC discocyte shape on RBC volume. The meanings of the links are described in the text. Because the volume affects the shape (dashed links) the described system as a whole represents a closed regulatory loop. It is indicated that Piezo1 Ca^++^ permeability can be affected either by membrane curvature or membrane lateral tension.

The essential ingredients of the proposed mechanism of the effect of Piezo1 on RBC volume are schematically represented by the cause-effect links shown in Figure 5. These links represent either experimental evidence or the results of theory or modeling. The upper dashed link indicates that RBC discocyte shape, according to theories described in the subsection “Interpretation of RBC and Vesicle Shapes on the Basis of Membrane Bending,” depends on RBC reduced volume. The lower dashed line link represents Eq.1. The link between RBC content of K^+^ and RBC volume is in a broad sense the consequence of the fact that RBC volume is established through osmotic equilibrium with the surrounding solution and that thus depends on the level of its cytoplasm cations. As discussed in section “RBC Volume and Related Aspects of the Variability of RBC Population” the regulation of cell cation content operates on the basis of active and passive membrane cation permeabilities (Hoffmann et al., 2009). The model concentrates on the homeostasis of K^+^. The preceding two links are thus based on the experiments of Cahalan et al. (2015) who have shown that Piezo1 channels act by the way that, when they are temporarily transformed into their open conformation, allow the influx calcium ions which activate the potassium specific Gárdos channels. The enhanced leakage of potassium ions then causes a decrease of their cell content and consequent loss of water. The Piezo1–Gárdos channel system is thus considered as a complement to the mechanism of RBC volume regulation based on the balance between influx and efflux of cations K^+^ and Na^+^. In the model it is assumed that, due to active Ca^++^ efflux, the Gárdos channels are on the average open only part of the time and that it is therefore reasonable to express membrane permeability coefficient for K^+^ (*P*_K_) as the sum two contributions

(7) P=KP+K,0fPGGK,

where *P*_K,G_ is RBC K^+^ permeability of its Gárdos channels, *f*_G_ the average fraction of them that are open, and *P*_K,0_ the potassium permeability of its other K^+^ channels. Due to osmotic equilibrium between its interior and exterior (see section “RBC Volume and Related Aspects of the Variability of RBC Population”), RBC volume is at larger values of *f*_G_ smaller. In the treated model we derived a relationship between *f*_G_ and the reduced volume *v* in which appeared as model parameters the ratio *P*_K,G_/*P*_K,0_, the relative amount of other RBC cytoplasm ingredients that cannot penetrate the membrane, and the reduced volume at *f*_G_ = 0. The crucial task of the model was to reveal a plausible mechanism for the effect of RBC shape on the fraction of time that Piezo1 channels are open. In the model it was proposed that there is another relationship between *f*_G_ and *v* based on the dependence of Piezo1 cation permeability on RBC shape. This relationship is represented in Figure 5 by the links that relate RBC discocyte shape and Piezo1 Ca^++^ permeability. The theory described in sub-section “Interpretation of RBC and Vesicle Shapes on the Basis of Membrane Bending” makes it possible to determine reduced mean membrane curvature (*h*) at each point on the membrane and its dependence on the reduced volume *v*. In the model (Svetina et al., 2019) it was shown that its value in RBC poles can be well represented by a linear function

(8) $$h_{\text{pole}}=h_{\mathrm{pole},r}+\beta_{\text{pole}}\,\left(v-v_{\text{r}}\right)$$

where *h*_pole,r_ is the reduced mean curvature at an arbitrarily chosen reference reduced volume *v*_r_. The value of the coefficient β_pole_ is 4.0. It was then taken into account that due to Piezo1 intrinsic curvature and its interaction with the membrane (Eq. 6), its molecules would tend to concentrate in the regions of RBC poles. On the basis of the assumption of that open Piezo1 conformation is less curved than its closed conformation it follows that, at the decrease of *v*, the probability that Piezo1 is closed increases. The parameters that defined thus obtained increasing function *f*_G_(*v*) are a combination of parameters that appear in Eqs. 6 and 8. Due to thus obtained relationships between *f*_G_ and *v* it is possible to express these two parameters in terms of other RBC structural parameters. On the basis of assuming the curvature dependent Piezo1–lipid matrix interaction it has been thus established that the system operates as a negative feedback regulatory loop between the average of the fraction of open Gárdos channels (*f*_G_) and the RBC reduced volume (*v*).

The described model was meant primarily to serve as the proof of principle for Piezo1 based regulation of RBC volume. Therefore it involves many simplifications of the real system. For example, it was restricted to K^+^ homeostasis and did not take into consideration possible concomitant changes of RBC Na^+^ content; the fraction of open Piezo1 channels was calculated as if they would all be located at the RBC poles; it was assumed that there are only two relevant Piezo1 conformations, etc. However, some of the model predictions are general in that they do not depend on its specific features. The main outcome of the effect of the RBC discoid shape on its volume is that it implies the existence of a closed regulatory loop for RBC volume regulation. The Piezo1–Gárdos channel system can be considered as a complement to the mechanism for the regulation of cell volume that operates on the basis of active and passive membrane cation permeabilities (Hoffmann et al., 2009). Within the mechanism of RBC volume regulation based on the balance between influx and efflux of cations K^+^ and Na^+^, Piezo1 acts at the level of K^+^ efflux. The membrane permeability coefficient for K^+^ involves a contribution that depends on the RBC reduced volume *v* and thus also on the membrane area *A*. The consequence of the regulation of *v* is the strong *V*–*A* correlation. Such correlation has been observed by simultaneous measurements of *V* and *A* in the RBC population (Canham and Burton, 1968; Gifford et al., 2003) and is evidenced by the steepness of the osmotic fragility curve (Figure 4B). Confirmation of this prediction of the model lies in the fact that the *V*–*A* correlation is lost, either in the absence of Piezo1 or by the application of the Ca^2+^ ionophore A23187 which overrules its action (Cahalan et al., 2015). The regulation of the RBC reduced volume is physiologically important because RBC, during the process of its aging, constantly releases nanovesicles (Westerman and Porter, 2016). In this type of vesiculation the loss of area is more significant than the loss of volume and, therefore, without this regulation the cells would lose their deformability because of the consequent increase of their reduced volume.

The model presented here points to the involvement of the RBC discoid shape in the fine regulation of its volume in a rather consistent manner. However, there are still many unanswered questions that require further experimentation. One such concern is whether the response of Piezo1 to change of RBC shape is due to change of membrane curvature or to the change of membrane lateral tension (Figure 5). For example, the theory of vesicle shapes predicts that the lateral tension is negative and that its absolute value at lowering the *v* increases (Svetina and Žekš, 1989). Changes of the lateral tension thus act in the appropriate direction but their effect was estimated to be small (Svetina et al., 2019). It could, however, be enhanced by the action of myosin motors (Smith et al., 2018). The dilemma about possible role of lateral tension could be resolved by experimental determination of the distribution of Piezo1 channels over the RBC membrane. Because mean principal membrane curvature is most negative in the RBC dimples, the analogously curved Piezo1 (Guo and MacKinnon, 2017) would preferentially reside in this region. With regard to the question as to whether the described mechanism of fine regulation of RBC volume also functions *in vivo* it should be realized that RBCs spend only about 50% of their time in veins, in which hydrodynamic conditions allow them to establish their discocyte shape. Namely, freely movable, membrane embedded proteins have diffusion coefficients that would cause them to equilibrate along the whole RBC surface in about 1 min. It would thus be important to determine whether the Piezo1 lateral diffusion coefficient is, for one reason or another, sufficiently smaller. It could be smaller because the RBC membrane is crowded (with more than 10^4^ proteins per 1 μm^2^) or because the Piezo1 molecule modifies the shape of the surrounding membrane to the size (Haselwandter and MacKinnon, 2018) that fits well into the area of the triangle of the structural unit of the hexagonal network of the spectrin skeleton. With regard to the Piezo1 oligomeric homo-trimer structure it can be noted that, in the case where its subunits can, independently, have two different conformation, it may attain at least four different structures, of which two have axial symmetry and two not. It still has to be established which of these structures corresponds to the Piezo1 open state. In the analyses of the variations within RBC population it has been assumed that there is no variation in membrane areal density of different RBC membrane proteins. In the context of the presented model it would be of particular interest to determine whether there are differences in the variability parameters of pumps and channels that are involved in the regulation of RBC volume.

There are also many aspects of the proposed model that require further theoretical modeling. For example, there is the question as to what is causing, in the *A*–*V* scatter plot, the remaining cell to cell variability. It could be ascribed to RBC variability with respect to its hemoglobin content (Svetina, 1982), but also to cell to cell variability of the ratio between the permeabilities of Gárdos channel and other potassium channels. In the model presented here it was taken that, for a given fixed membrane shape, the inclusions redistribute due to their interaction with the surrounding membrane. However, in general the effect is mutual: due to the curvature dependent interaction of Piezo1 molecules with the surrounding membrane, the RBC shape may also change (Božič et al., 2006). The consequent coupling between the RBC shape and the conformational state of Piezo1 molecules could cause oscillatory non-stationary behavior of the treated system. One has to be aware also of possible second order factors such as membrane lateral inhomogeneity (Hoffman, 2019). It still needs to be established as to the nature of the physical basis for the Piezo1–lipid matrix interaction. It could be based on the perturbed energy of the lipid bilayer (Haselwandter and MacKinnon, 2018) but may also involve specific interactions between a protein and its surrounding molecules, e.g., the curvature dependence of the number of hydrogen bonds that Piezo1 forms with surrounding lipids. Curvature dependent Piezo1–lipid matrix interaction may also involve energy terms due to Piezo1 intrinsic elasticity (Lin et al., 2019).

## Author Contributions

The author confirms being the sole contributor of this work and has approved it for publication.

## Conflict of Interest

The author declares that the research was conducted in the absence of any commercial or financial relationships that could be construed as a potential conflict of interest.

## References

[B1] Armstrong C. M. (2003). The Na/K pump, Cl ion, and osmotic stabilization of cells. *Proc. Natl. Acad. Sci. U.S.A.* 100 6257–6262. 10.1073/pnas.0931278100 12730376 PMC156359

[B2] Ataullakhanov F. I. Korunova N. O. Spiridonov I. S. Pivovarov I. O. Kalyagina N. V. Martinov M. V. (2009). How erythrocyte volume is regulated, or what mathematical models can and cannot do for biology. *Biochem. Mosc. Suppl. Ser. A Membr. Cell Biol.* 3 101–115. 10.1134/s1990747809020019

[B3] Božič B. Kralj-Iglič V. Svetina S. (2006). Coupling between vesicle shape and lateral distribution of mobile membrane inclusions. *Phys. Rev. E* 73:041915. 16711844 10.1103/PhysRevE.73.041915

[B4] Brumen M. Glaser R. Svetina S. (1979). Osmotic states of red blood cells. *Bioelectrochem. Bioenerget.* 6 227–241. 10.1016/0302-4598(79)87010-5

[B5] Brumen M. Glaser R. Svetina S. (1981). Study of the red blood cell osmotic behaviour in the “pump-leak” model. *Period. Biol.* 83 151–153.

[B6] Cahalan S. M. Lukacs V. Ranade S. S. Chien S. Bandell M. Patapoutian A. (2015). Piezo1 links mechanical forces to red blood cell volume. *eLife* 4:e07370. 10.7554/eLife.07370 26001274 PMC4456639

[B7] Canham P. B. (1970). Minimum energy of bending as a possible explanation of biconcave shape of human red blood cell. *J. Theor. Biol.* 26 61–81. 10.1016/s0022-5193(70)80032-7 5411112

[B8] Canham P. B. Burton A. C. (1968). Distribution of size and shape in populations of normal human red cells. *Circ. Res.* 22 405–422. 10.1161/01.res.22.3.405 5639051

[B9] Cinar E. Zhou S. DeCourcey J. Wang Y. Waugh R. E. Wan J. (2015). Piezo1 regulates mechanotransductive release of ATP from human RBCs. *Proc. Natl. Acad. Sci. U.S.A.* 112 11783–11788. 10.1073/pnas.1507309112 26351678 PMC4586865

[B10] Coste B. Mathur J. Schmidt M. Earley T. J. Ranade S. Petrus M. J. (2010). Piezo1 and Piezo2 are essential components of distinct mechanically activated cation channels. *Science* 330 55–60. 10.1126/science.1193270 20813920 PMC3062430

[B11] Danielczok J. G. Terriac E. Hertz L. Petkova-Kirova P. Lautenschläger F. Laschke M. W. (2017). Red blood cell passage of small capillaries is associated with transient Ca2+-mediated adaptations. *Front. Physiol.* 8:979. 10.3389/fphys.2017.00979 29259557 PMC5723316

[B12] Deuling H. J. Helfrich W. (1976). Red blood cell shapes as explained on the basis of curvature elasticity. *Biophys. J.* 71 861–868. 10.1016/s0006-3495(76)85736-0 938726 PMC1334911

[B13] Discher D. E. Boal D. H. Boey S. K. (1998). Simulations of the erythrocyte skeleton at large deformation. II. Micropipette aspiration. *Biophys. J.* 75 1584–1597. 10.1016/s0006-3495(98)74076-7 9726959 PMC1299832

[B14] Discher D. E. Mohandas N. (1996). Kinematics of red cell aspiration by fluorescence-imaged microdeformation. *Biophys. J.* 71 1680–1694. 10.1016/s0006-3495(96)79424-9 8889146 PMC1233638

[B15] Discher D. E. Mohandas N. Evans E. A. (1994). Molecular maps of red cell deformation: hidden elasticity and in situ connectivity. *Science* 266 1032–1035. 10.1126/science.7973655 7973655

[B16] Evans E. A. (1980). Minimum energy analysis of membrane deformation applied to pipet aspiration and surface adhesion of red blood cells. *Biophys. J.* 30 265–284. 10.1016/s0006-3495(80)85093-4 7260275 PMC1328733

[B17] Evans E. A. Waugh R. Melnik L. (1976). Elastic area compressibility modulus of red cell membrane. *Biophys. J.* 16 585–595. 10.1016/s0006-3495(76)85713-x 1276386 PMC1334882

[B18] Fedosov D. A. Caswell B. Karniadakis G. E. (2010). Systematic coarse-graining of spectrin-level red blood cell models. *Comput. Methods Appl. Mech. Eng.* 199 1937–1948. 10.1016/j.cma.2010.02.001 24353352 PMC3864857

[B19] Freedman J. C. Hoffman J. F. (1979). Ionic and osmotic equilibria of human red blood cells treated by nystatin. *J. Gen. Physiol.* 74 157–185. 10.1085/jgp.74.2.157 490141 PMC2228501

[B20] Ge J. Li W. Zhao Q. Chen M. Zhi P. Li R. (2015). Architecture of the mammalian mechanosensitive Piezo1 channel. *Nature* 527 64–69. 10.1038/nature15247 26390154

[B21] Gifford S. C. Frank M. G. Derganc J. Gabel C. Austin R. H. Yoshida T. (2003). Parallel microchannel-based measurements of individual erythrocyte areas and volumes. *Biophys. J.* 84 623–633. 10.1016/s0006-3495(03)74882-6 12524315 PMC1302643

[B22] Goldstein R. E. (2018). Are theoretical results ‘results’? *eLife* 7:e40018. 10.7554/eLife.40018 PMC6056240 30033910

[B23] Guo Y. R. MacKinnon R. (2017). Structure-based membrane dome mechanism for Piezo mechanosensitivity. *eLife* 6:e33660. 10.7554/eLife.33660 29231809 PMC5788504

[B24] Haselwandter C. A. MacKinnon R. (2018). Piezo’s membrane footprint and its contribution to mechanosensitivity. *eLife* 7:e41968. 10.7554/eLife.41968 30480546 PMC6317911

[B25] Heinrich V. Svetina S. Žekš B. (1993). Nonaxisymmetric vesicle shapes in a generalized bilayer-couple model and the transition between oblate and prolate axisymmetric shapes. *Phys. Rev. E* 48 3112–3123. 10.1103/physreve.48.3112 9960950

[B26] Helfrich W. (1973). Elastic properties of lipid bilayers: theory and possible experiments. *Z. Naturforsch.* 28c 693–703. 10.1515/znc-1973-11-1209 4273690

[B27] Hoffman J. F. (2019). Reflections on the crooked timber of red blood cell physiology. *Blood Cells Mol. Dis.* 79:102354. 10.1016/j.bcmd.2019.102354 31449971

[B28] Hoffmann E. K. Lambert I. H. Pedersen S. F. (2009). Physiology of cell volume regulation in vertebrates. *Physiol. Rev.* 89 193–277. 10.1152/physrev.00037.2007 19126758

[B29] Hwang W. C. Waugh R. E. (1997). Energy of dissociation of lipid bilayer from the membrane skeleton of red blood cells. *Biophys. J.* 72 2669–2678. 10.1016/s0006-3495(97)78910-0 9168042 PMC1184464

[B30] Kay A. R. Blaustein M. P. (2019). Evolution of our understanding of cell volume regulation by the pump-leak mechanism. *J. Gen. Physiol.* 151 407–416. 10.1085/jgp.201812274 30782603 PMC6445581

[B31] Kralj-Iglič V. Heinrich V. Svetina S. Žekš B. (1999). Free energy of closed membrane with anisotropic inclusions. *Eur. Phys. J. B* 10 5–8. 10.1007/s100510050822

[B32] Kralj-Iglič V. Svetina S. Žekš B. (1996). Shapes of bilayer vesicles with membrane embedded molecules. *Eur. Biophys. J.* 24 311–321. 10.1007/bf00180372 8766690

[B33] Kuchel P. W. Shishmarev D. (2017). Accelerating metabolism and transmembrane cation flux by distorting red blood cells. *Sci. Adv.* 3:eaao1016. 10.1126/sciadv.aao1016 29057326 PMC5647125

[B34] Lanotte L. Mauer J. Mendez S. Fedosov D. A. Fromental J.-M. Claveria V. (2016). Red cells’ dynamic morphologies govern blood shear thinning under microcirculatory flow conditions. *Proc. Natl. Acad. Sci. U.S.A.* 113 13289–13294. 10.1073/pnas.1608074113 27834220 PMC5127344

[B35] Lew V. L. Bookchin R. M. (1986). Volume, pH, and ion-content regulation in human red cells: analysis of transient behavior with an integrated model. *J. Membr. Biol.* 92 57–74. 10.1007/bf01869016 3746891

[B36] Lew V. L. Raftos J. E. Sorette M. Bookchin R. M. Mohandas N. (1995). Generation of normal human red cell volume, hemoglobin content, and membrane area distributions by “birth” or regulation? *Blood* 86 334–341. 10.1182/blood.v86.1.334.bloodjournal861334 7795242

[B37] Lin Y. C. Guo R. Miyagi A. Levring J. MacKinnon R. Scheuring S. (2019). Force-induced conformational changes in PIEZO1. *Nature* 92 57–74. 10.1038/s41586-019-1499-2 PMC7258172 31435018

[B38] Lux S. E. (2016). Anatomy of the red cell membrane skeleton: unanswered questions. *Blood* 127 187–199. 10.1182/blood-2014-12-512772 26537302

[B39] McMahon H. T. Gallop J. L. (2005). Membrane curvature and mechanisms of dynamic cell membrane remodelling. *Nature* 438 590–596. 10.1038/nature04396 16319878

[B40] Miao L. Seifert U. Wortis M. Döbereiner H.-G. (1994). Budding transitions of fluid-bilayer vesicles: the effect of area-difference elasticity. *Phys. Rev. E* 49 5389–5407. 10.1103/physreve.49.5389 9961866

[B41] Mohandas N. Chasis J. A. (1993). Red blood cell deformability, membrane material properties and shape: regulation by transmembrane, skeletal and cytosolic proteins and lipids. *Semin. Hematol.* 30 171–192. 8211222

[B42] Mohandas N. Evans E. (1994). Mechanical properties of the red cell membrane in relation to molecular structure and genetic effects. *Ann. Rev. Biophys. Biomol. Struct.* 23 787–818. 10.1146/annurev.bb.23.060194.004035 7919799

[B43] Mohandas N. Gallagher P. G. (2008). Red cell membrane: past, present, and future. *Blood* 112 3939–3947. 10.1182/blood-2008-07-161166 18988878 PMC2582001

[B44] Mukhopadhyay R. Lim G. H. W. Wortis M. (2002). Echinocyte shapes: bending, stretching, and shear determine spicule shape and spacing. *Biophys. J.* 82 1756–1772. 10.1016/s0006-3495(02)75527-6 11916836 PMC1301974

[B45] Murthy S. E. Dubin A. E. Patapoutian A. (2017). Piezos thrive under pressure: mechanically activated ion channels in health and disease. *Nat. Rev. Mol. Cell Biol.* 18 771–783. 10.1038/nrm.2017.92 28974772

[B46] Peng Z. Asaro R. J. Zhu Q. (2010). Multiscale simulation of erythrocyte membranes. *Phys. Rev. E* 81:031904. 20365767 10.1103/PhysRevE.81.031904 PMC2876725

[B47] Raphael R. M. Waugh R. E. (1996). Accelerated interleaflet transport of phosphatidylcholine molecules in membranes under deformation. *Biophys. J.* 82 1756–1772. 8874013 10.1016/S0006-3495(96)79340-2 PMC1233606

[B48] Saotome K. Murthy S. E. Kefauver J. M. Whitwam T. Patapoutian A. Ward A. B. (2018). Structure of the mechanically activated ion channel Piezo1. *Nature* 554 481–486. 10.1038/nature25453 29261642 PMC6010196

[B49] Seifert U. (1997). Configurations of fluid membranes and vesicles. *Adv. Phys.* 46 13–137. 10.1080/00018739700101488

[B50] Seifert U. Berndl K. Lipowsky R. (1991). Shape transformations of vesicles - phase-diagram for spontaneous-curvature and bilayer-coupling models. *Phys. Rev. A* 44 1182–1202. 10.1103/physreva.44.1182 9906067

[B51] Sheetz M. P. Singer S. J. (1974). Biological-membranes as bilayer couples - molecular mechanism of drug-erythrocyte interactions. *Proc. Natl. Acad. Sci. U.S.A.* 71 4457–4461. 10.1073/pnas.71.11.4457 4530994 PMC433905

[B52] Smith A. S. Nowak R. B. Zhou S. Giannetto M. Gokhin D. S. Papoin J. (2018). Myosin IIA interacts with the spectrin-actin membrane skeleton to control red blood cell membrane curvature and deformability. *Proc. Natl. Acad. Sci. U.S.A.* 115 E4377–E4385. 29610350 10.1073/pnas.1718285115 PMC5948966

[B53] Švelc T. Svetina S. (2012). Stress-free state of the red blood cell membrane and the deformation of its skeleton. *Cell. Mol. Biol. Lett.* 17 217–227. 10.2478/s11658-012-0005-8 22302416 PMC6275672

[B54] Svetina S. (1982). Relations among variations in human red cell volume, density, membrane area, hemoglobin content and cation content. *J. Theor. Biol.* 95 123–134. 10.1016/0022-5193(82)90291-0 7087492

[B55] Svetina S. (1998). Skeleton –bilayer interaction and the shape of red blood cells. *Cell. Mol. Biol. Lett.* 3 449–463.

[B56] Svetina S. (2015). Curvature–dependent protein–lipid bilayer interaction and cell mechanosensitivity. *Eur. Biophys. J.* 44 513–519. 10.1007/s00249-015-1046-5 26033539

[B57] Svetina S. (2017). Investigating cell functioning by theoretical analysis of cell-to-cell variability. *Eur. Biophys. J.* 46 739–748. 10.1007/s00249-017-1258-y 28986665

[B58] Svetina S. Brumen M. Gros M. Vrhovec S. Žnidarčič T. (2003). “On the variation of parameters that characterize the state of a physiological system. Red blood cells as an example,” in *Simulations in Biomedicine V*, eds Arnež Z. M. Brebbia C. A. Solina F. Stankovski V. (Southampton: WITT Press), 3–14.

[B59] Svetina S. Iglič A. Kralj-Iglič V. Žekš B. (1996). Cytoskeleton and red cell shape. *Cell. Mol. Biol. Lett.* 1 67–78.

[B60] Svetina S. Kokot G. Švelc Kebe T. Žekš B. Waugh R. E. (2016). A novel strain energy relationship for red blood cell membrane skeleton based on spectrin stiffness and its application to micropipette deformation. *Biomech. Model. Mechanobiol.* 15 745–758. 10.1007/s10237-015-0721-x 26376642 PMC4794432

[B61] Svetina S. Kralj-Iglič V. Žekš B. (1990). “Cell shape and lateral distribution of mobile membrane constituents,” in *Biophysics of Membrane Transport: Tenth School on Biophysics of Membrane Transport, Part II*, eds Kuczera J. Przestalski S. (Wrocław: Agricultural University of Wrocław), 139–155.

[B62] Svetina S. Kuzman D. Waugh R. E. Ziherl P. Žekš B. (2004). The cooperative role of membrane skeleton and bilayer in the mechanical behaviour of red blood cells. *Bioelectrochemistry* 62 107–113. 10.1016/j.bioelechem.2003.08.002 15039011

[B63] Svetina S. Ottova-Leitmannová A. Glaser R. (1982). Membrane bending energy relation to bilayer couples concept of red blood cell shape transformations. *J. Theor. Biol.* 94 13–23. 10.1016/0022-5193(82)90327-7 7078204

[B64] Svetina S. Švelc Kebe T. Božič B. (2019). A model of Piezo1 based regulation of red blood cell volume. *Biophys. J.* 116 151–164. 10.1016/j.bpj.2018.11.3130 30580922 PMC6342734

[B65] Svetina S. Žekš B. (1983). Bilayer couple hypothesis of red-cell shape transformations and osmotic hemolysis. *Biomed. Biochim. Acta* 42 S86–S90. 6675721

[B66] Svetina S. Žekš B. (1989). Membrane bending energy and shape determination of phospholipid vesicles and red blood cells. *Eur. Biophys. J.* 17 101–111. 10.1007/bf00257107 2766997

[B67] Svetina S. Žekš B. (1990). The mechanical behavior of cell membranes as a possible physical origin of cell polarity. *J. Theor. Biol.* 146 115–122. 10.1016/s0022-5193(05)80047-5 2232827

[B68] Svetina S. Žekš B. (1996). “Elastic properties of closed bilayer membranes and the shapes of giant phospholipid vesicles,” in *Handbook of Nonmedical Applications of Liposomes*, Vol.I, eds Lasic D. D. Barenholz Y. (Boca Raton, FL: CRC), 13–42.

[B69] Svetina S. Žekš B. (2014). Nonlocal membrane bending: a reflection, the facts and its relevance. *Adv. Coll. Interface Sci.* 208 189–196. 10.1016/j.cis.2014.01.010 24529971

[B70] Svetina S. Žekš B. Waugh R. E. Raphael R. M. (1998). Theoretical analysis of the effect of the transbilayer movement of phospholipid molecules on the dynamic behavior of a microtube pulled out of an aspirated vesicle. *Eur. Biophys. J.* 27 197–209. 10.1007/s002490050126 9615393

[B71] Tomishige M. Sako Y. Kusumi A. (1998). Regulation mechanism of the lateral diffusion of band 3 in erythrocyte membranes by the membrane skeleton. *J. Cell Biol.* 142 989–1000. 10.1083/jcb.142.4.989 9722611 PMC2132872

[B72] Tosteson D. C. Hoffman J. F. (1960). Regulation of cell volume by active cation transport in high and low potassium sheep red cells. *J. Gen. Physiol.* 44 169–196. 13777653 10.1085/jgp.44.1.169 PMC2195084

[B73] Westerman M. Porter J. B. (2016). Red blood cell-derived microparticles: an overview. *Blood Cells Mol. Dis.* 59 134–139. 10.1016/j.bcmd.2016.04.003 27282583

[B74] Zarychanski R. Schulz V. P. Brett L. H. Maksimova Y. Houston D. S. Smith J. (2012). Mutations in the mechanotransduction protein PIEZO1 are associated with hereditary xerocytosis. *Blood* 120 1908–1915. 10.1182/blood-2012-04-422253 22529292 PMC3448561

[B75] Zhao Q. Zhou H. Chi S. Wang Y. Wang J. Geng J. (2018). Structure and mechanogating mechanism of the Piezo1 channel. *Nature* 554 487–492. 10.1038/nature25743 29469092

[B76] Zimmerberg J. Kozlov M. M. (2006). How proteins produce cellular membrane curvature. *Nat. Rev. Mol. Cell Biol.* 7 9–19. 16365634 10.1038/nrm1784

